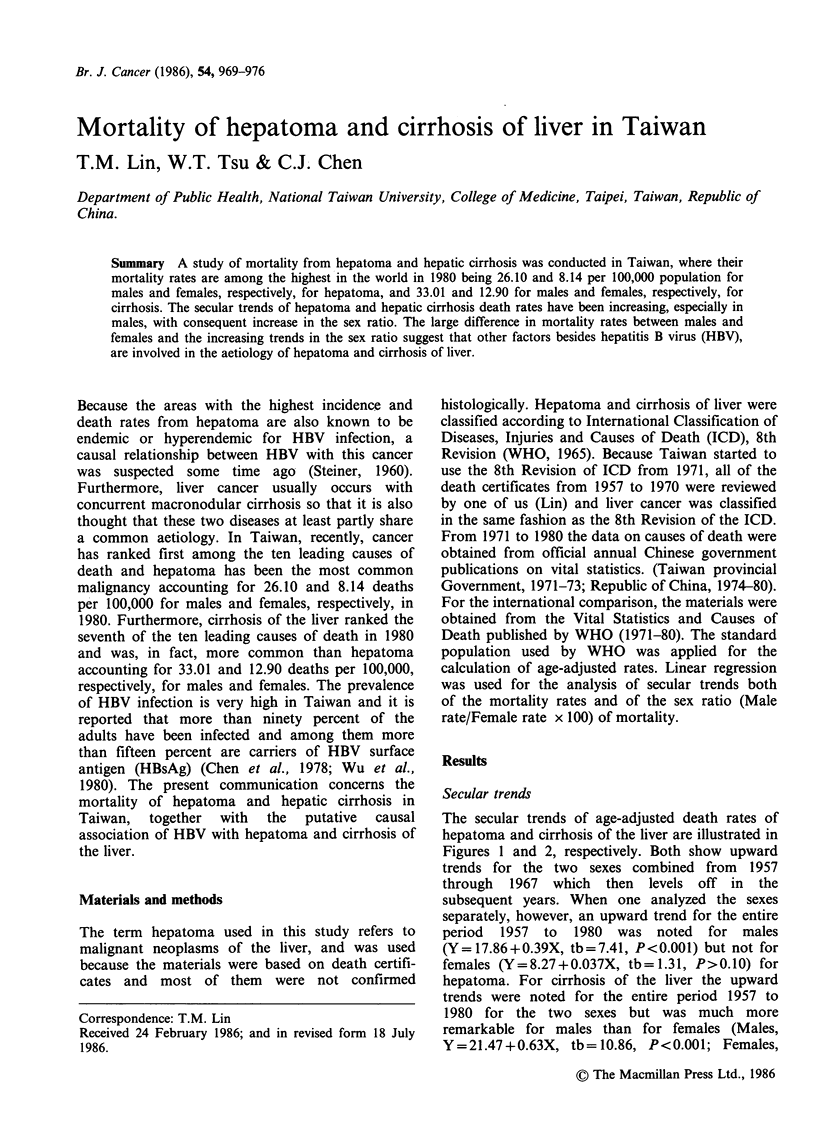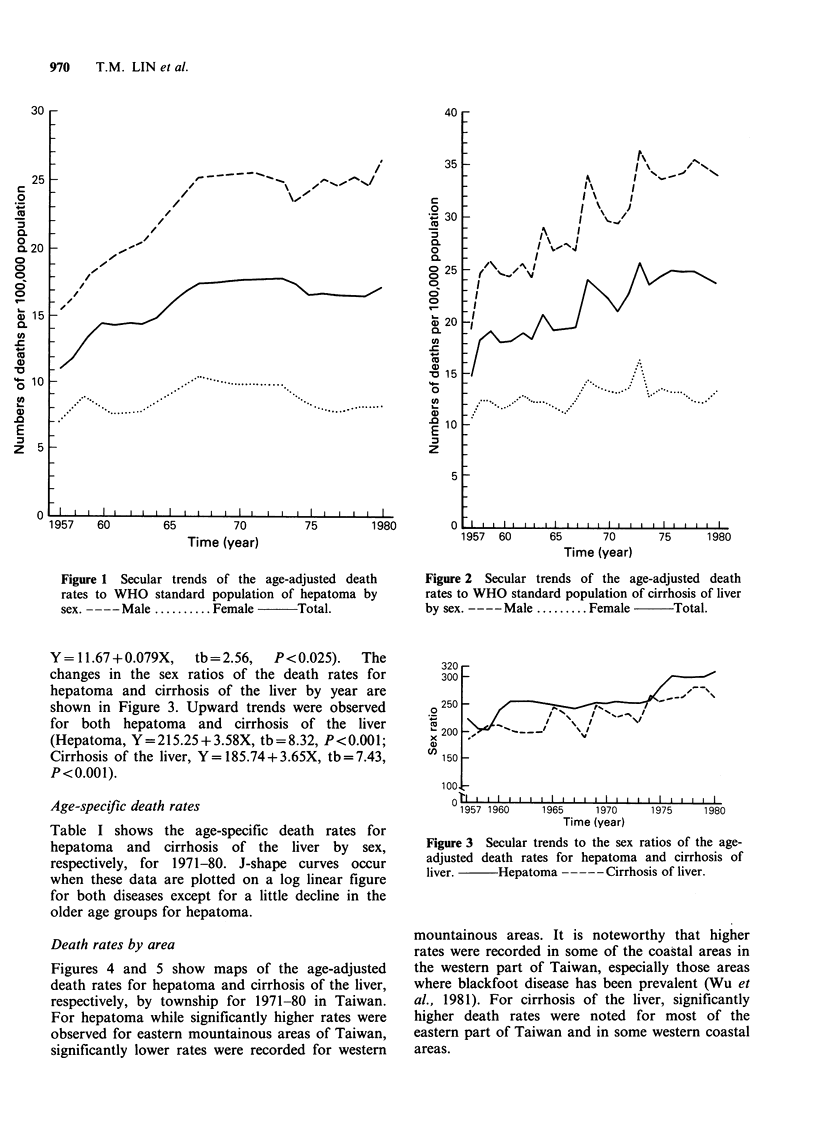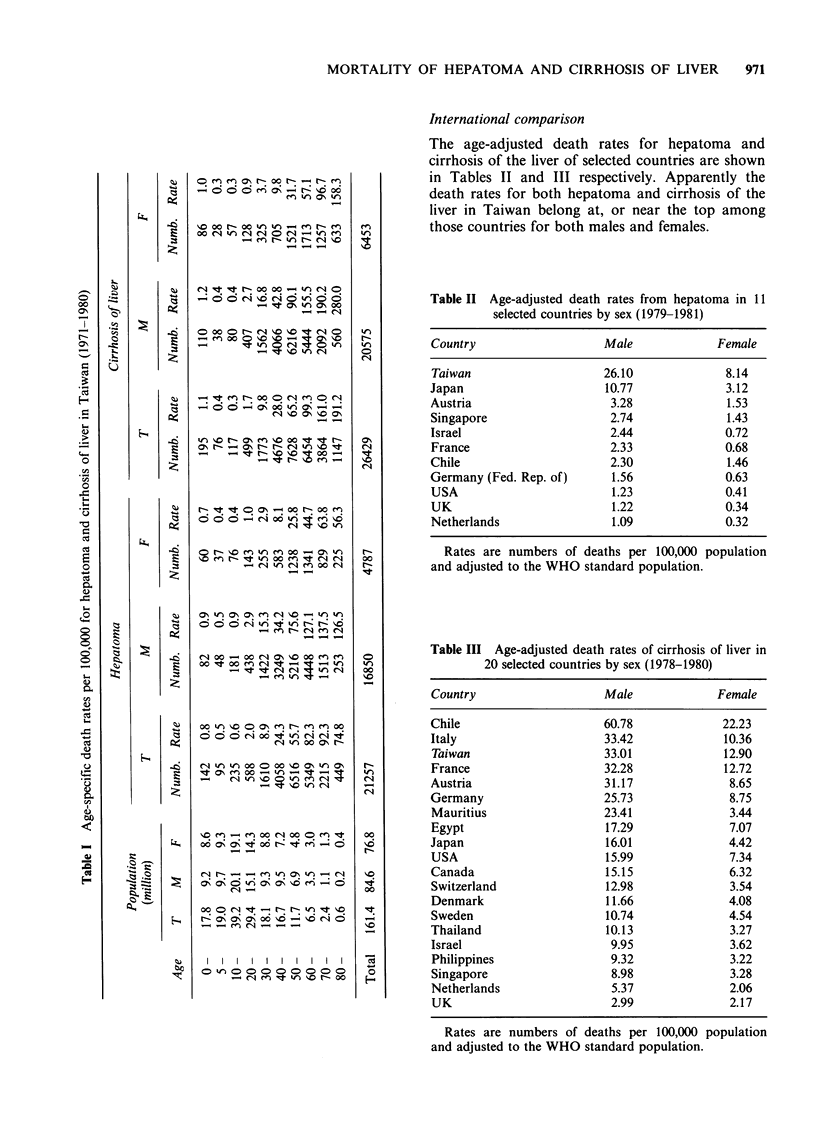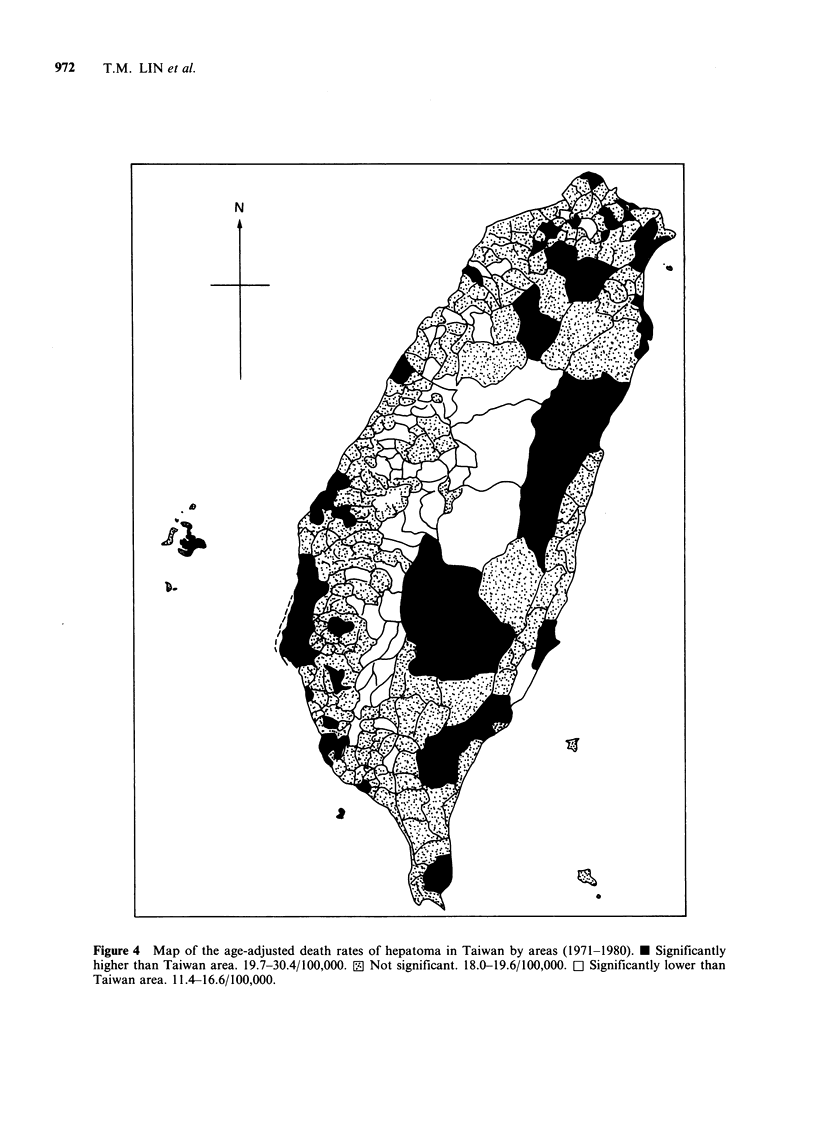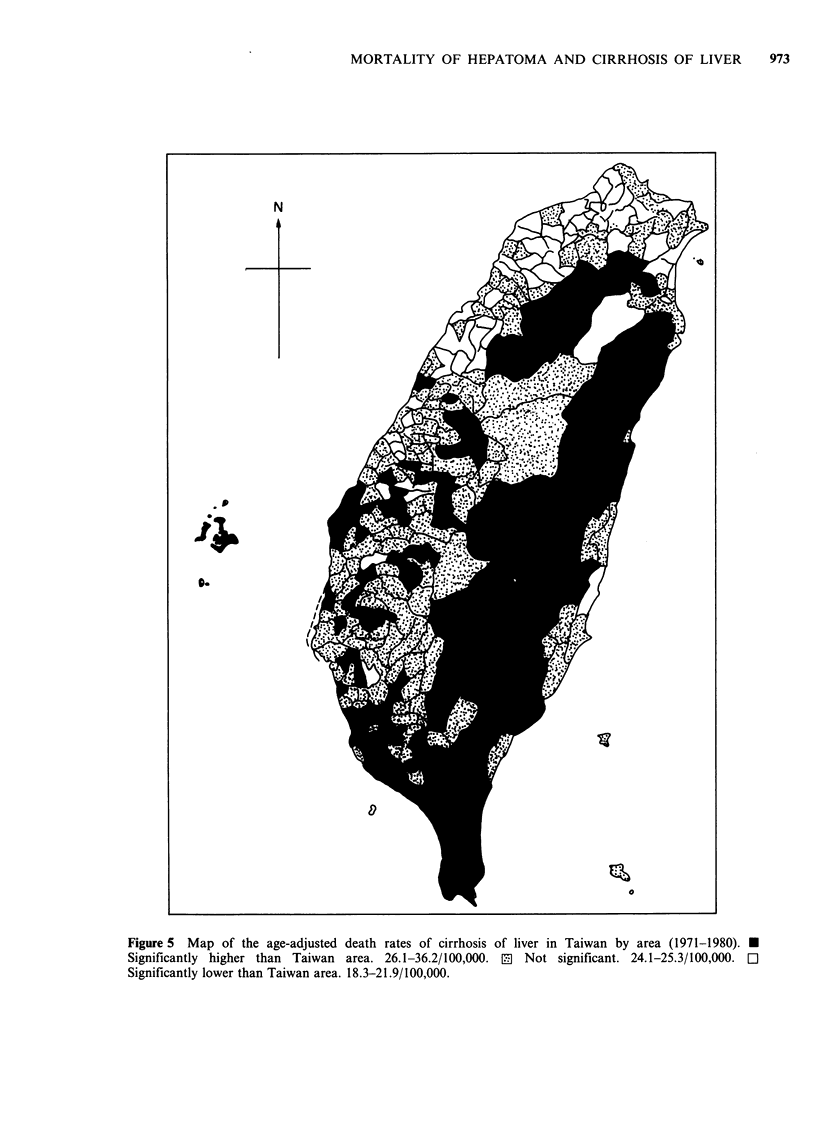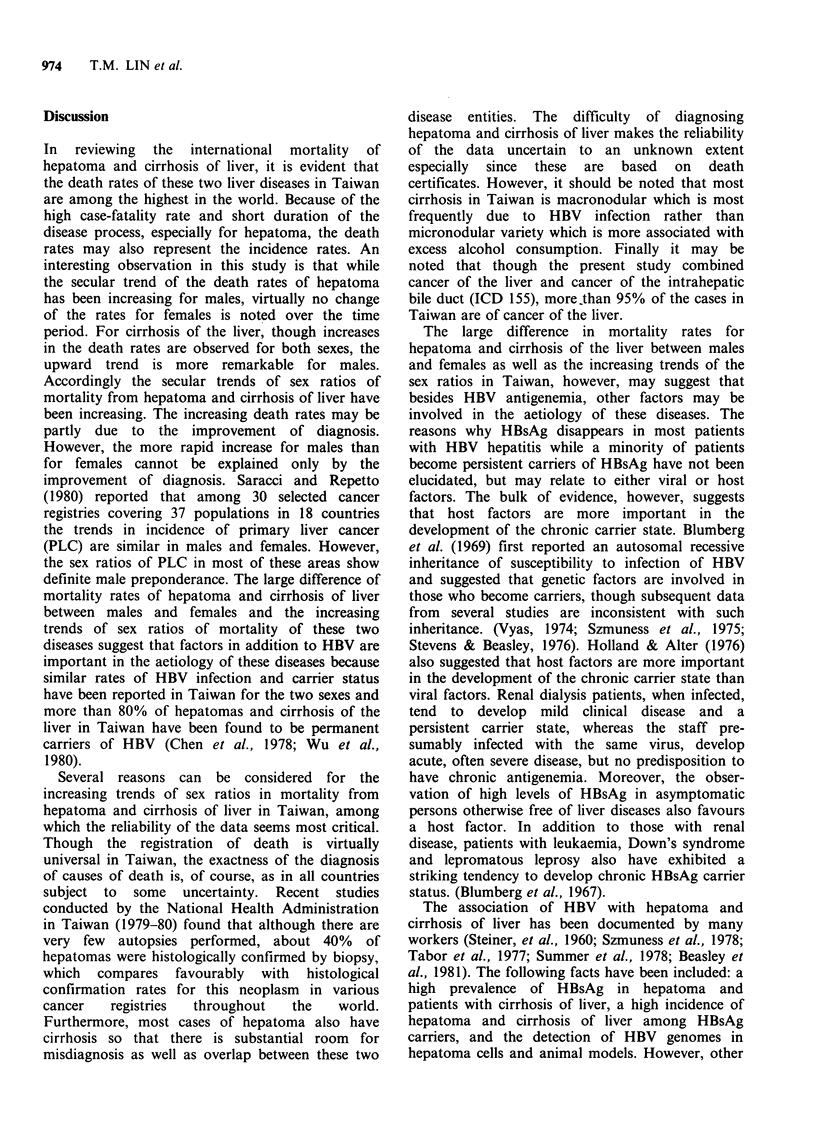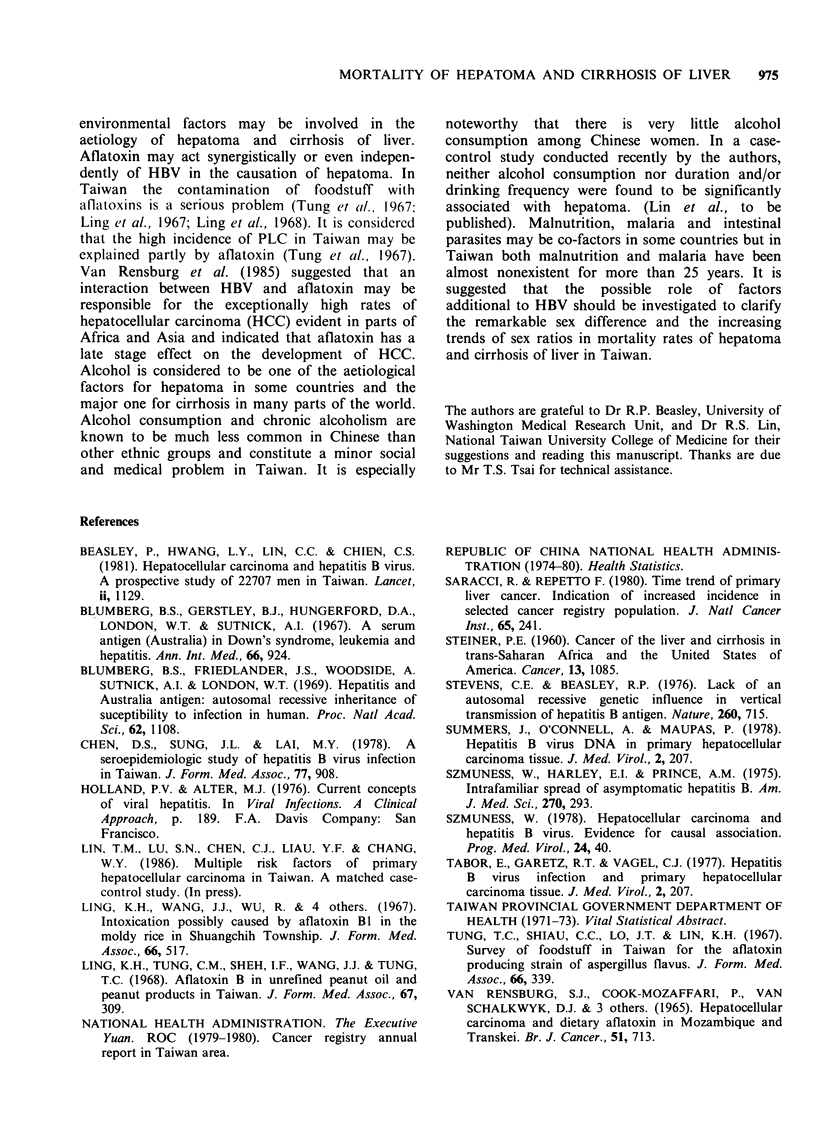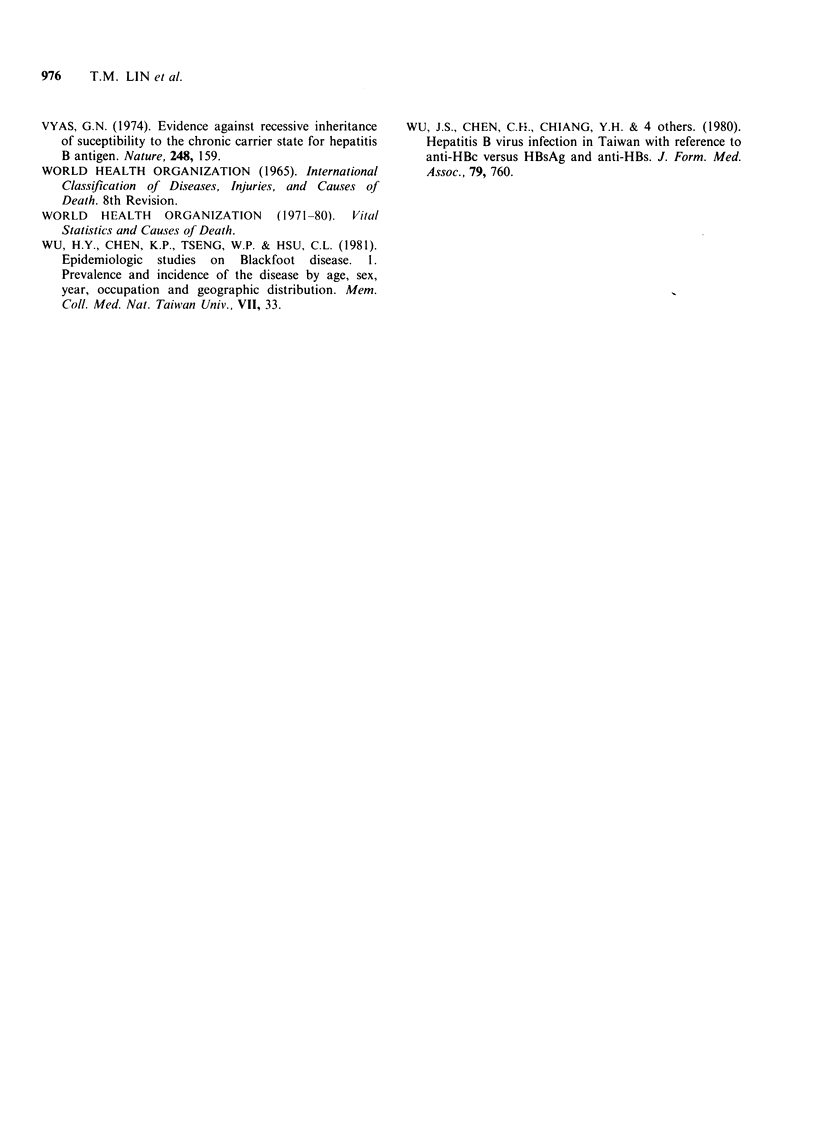# Mortality of hepatoma and cirrhosis of liver in Taiwan.

**DOI:** 10.1038/bjc.1986.269

**Published:** 1986-12

**Authors:** T. M. Lin, W. T. Tsu, C. J. Chen

## Abstract

A study of mortality from hepatoma and hepatic cirrhosis was conducted in Taiwan, where their mortality rates are among the highest in the world in 1980 being 26.10 and 8.14 per 100,000 population for males and females, respectively, for hepatoma, and 33.01 and 12.90 for males and females, respectively, for cirrhosis. The secular trends of hepatoma and hepatic cirrhosis death rates have been increasing, especially in males, with consequent increase in the sex ratio. The large difference in mortality rates between males and females and the increasing trends in the sex ratio suggest that other factors besides hepatitis B virus (HBV), are involved in the aetiology of hepatoma and cirrhosis of liver.


*Br. J. Cancer* (1986), **54**, 969–976

### Mortality of hepatoma and cirrhosis of liver in Taiwan

T.M. Lin, W.T. Tsu & C.J. Chen

*Department of Public Health, National Taiwan University, College of Medicine, Taipei, Taiwan, Republic of China.*

**Summary** A study of mortality from hepatoma and hepatic cirrhosis was conducted in Taiwan, where their mortality rates are among the highest in the world in 1980 being 26.10 and 8.14 per 100,000 population for males and females, respectively, for hepatoma, and 33.01 and 12.90 for males and females, respectively, for cirrhosis. The secular trends of hepatoma and hepatic cirrhosis death rates have been increasing, especially in males, with consequent increase in the sex ratio. The large difference in mortality rates between males and females and the increasing trends in the sex ratio suggest that other factors besides hepatitis B virus (HBV), are involved in the aetiology of hepatoma and cirrhosis of liver.

Because the areas with the highest incidence and death rates from hepatoma are also known to be endemic or hyperendemic for HBV infection, a causal relationship between HBV with this cancer was suspected some time ago (Steiner, 1960). Furthermore, liver cancer usually occurs with concurrent macronodular cirrhosis so that it is also thought that these two diseases at least partly share a common aetiology. In Taiwan, recently, cancer has ranked first among the ten leading causes of death and hepatoma has been the most common malignancy accounting for 26.10 and 8.14 deaths per 100,000 for males and females, respectively, in 1980. Furthermore, cirrhosis of the liver ranked the seventh of the ten leading causes of death in 1980 and was, in fact, more common than hepatoma accounting for 33.01 and 12.90 deaths per 100,000, respectively, for males and females. The prevalence of HBV infection is very high in Taiwan and it is reported that more than ninety percent of the adults have been infected and among them more than fifteen percent are carriers of HBV surface antigen (HBsAg) (Chen *et al.*, 1978; Wu *et al.*, 1980). The present communication concerns the mortality of hepatoma and hepatic cirrhosis in Taiwan, together with the putative causal association of HBV with hepatoma and cirrhosis of the liver.

### Materials and methods

The term hepatoma used in this study refers to malignant neoplasms of the liver, and was used because the materials were based on death certificates and most of them were not confirmed histologically. Hepatoma and cirrhosis of liver were classified according to International Classification of Diseases, Injuries and Causes of Death (ICD), 8th Revision (WHO, 1965). Because Taiwan started to use the 8th Revision of ICD from 1971, all of the death certificates from 1957 to 1970 were reviewed by one of us (Lin) and liver cancer was classified in the same fashion as the 8th Revision of the ICD. From 1971 to 1980 the data on causes of death were obtained from official annual Chinese government publications on vital statistics. (Taiwan provincial Government, 1971–73; Republic of China, 1974–80). For the international comparison, the materials were obtained from the Vital Statistics and Causes of Death published by WHO (1971–80). The standard population used by WHO was applied for the calculation of age-adjusted rates. Linear regression was used for the analysis of secular trends both of the mortality rates and of the sex ratio (Male rate/Female rate $\times 100$) of mortality.

Correspondence: T.M. Lin
Received 24 February 1986; and in revised form 18 July 1986.

### Results

### *Secular trends*

The secular trends of age-adjusted death rates of hepatoma and cirrhosis of the liver are illustrated in Figures 1 and 2, respectively. Both show upward trends for the two sexes combined from 1957 through 1967 which then levels off in the subsequent years. When one analyzed the sexes separately, however, an upward trend for the entire period 1957 to 1980 was noted for males ($Y=17.86+0.39X$, $tb=7.41$, $P<0.001$) but not for females ($Y=8.27+0.037X$, $tb=1.31$, $P>0.10$) for hepatoma. For cirrhosis of the liver the upward trends were noted for the entire period 1957 to 1980 for the two sexes but was much more remarkable for males than for females (Males, $Y=21.47+0.63X$, $tb=10.86$, $P<0.001$; Females,

© The Macmillan Press Ltd., 1986

**Figure 1** Secular trends of the age-adjusted death rates to WHO standard population of hepatoma by sex. ---- Male .......... Female ——— Total.

**Figure 2** Secular trends of the age-adjusted death rates to WHO standard population of cirrhosis of liver by sex. ---- Male ......... Female ——— Total.

$Y = 11.67 + 0.079X$, $tb = 2.56$, $P < 0.025$). The changes in the sex ratios of the death rates for hepatoma and cirrhosis of the liver by year are shown in Figure 3. Upward trends were observed for both hepatoma and cirrhosis of the liver (Hepatoma, $Y = 215.25 + 3.58X$, $tb = 8.32$, $P < 0.001$; Cirrhosis of the liver, $Y = 185.74 + 3.65X$, $tb = 7.43$, $P < 0.001$).

*Age-specific death rates*

Table I shows the age-specific death rates for hepatoma and cirrhosis of the liver by sex, respectively, for 1971–80. J-shape curves occur when these data are plotted on a log linear figure for both diseases except for a little decline in the older age groups for hepatoma.

*Death rates by area*

Figures 4 and 5 show maps of the age-adjusted death rates for hepatoma and cirrhosis of the liver, respectively, by township for 1971–80 in Taiwan. For hepatoma while significantly higher rates were observed for eastern mountainous areas of Taiwan, significantly lower rates were recorded for western mountainous areas. It is noteworthy that higher rates were recorded in some of the coastal areas in the western part of Taiwan, especially those areas where blackfoot disease has been prevalent (Wu *et al.*, 1981). For cirrhosis of the liver, significantly higher death rates were noted for most of the eastern part of Taiwan and in some western coastal areas.

**Figure 3** Secular trends to the sex ratios of the age-adjusted death rates for hepatoma and cirrhosis of liver. ——— Hepatoma ----- Cirrhosis of liver.

**Table I** Age-specific death rates per 100,000 for hepatoma and cirrhosis of liver in Taiwan (1971–1980)

| | *Population (million)* | | | *Hepatoma* | | | | | | *Cirrhosis of liver* | | | | | |
|---|---|---|---|---|---|---|---|---|---|---|---|---|---|---|---|
| | | | | *T* | | *M* | | *F* | | *T* | | *M* | | *F* | |
| *Age* | *T* | *M* | *F* | *Numb.* | *Rate* | *Numb.* | *Rate* | *Numb.* | *Rate* | *Numb.* | *Rate* | *Numb.* | *Rate* | *Numb.* | *Rate* |
| 0 – | 17.8 | 9.2 | 8.6 | 142 | 0.8 | 82 | 0.9 | 60 | 0.7 | 195 | 1.1 | 110 | 1.2 | 86 | 1.0 |
| 5 – | 19.0 | 9.7 | 9.3 | 95 | 0.5 | 48 | 0.5 | 37 | 0.4 | 76 | 0.4 | 38 | 0.4 | 28 | 0.3 |
| 10 – | 39.2 | 20.1 | 19.1 | 235 | 0.6 | 181 | 0.9 | 76 | 0.4 | 117 | 0.3 | 80 | 0.4 | 57 | 0.3 |
| 20 – | 29.4 | 15.1 | 14.3 | 588 | 2.0 | 438 | 2.9 | 143 | 1.0 | 499 | 1.7 | 407 | 2.7 | 128 | 0.9 |
| 30 – | 18.1 | 9.3 | 8.8 | 1610 | 8.9 | 1422 | 15.3 | 255 | 2.9 | 1773 | 9.8 | 1562 | 16.8 | 325 | 3.7 |
| 40 – | 16.7 | 9.5 | 7.2 | 4058 | 24.3 | 3249 | 34.2 | 583 | 8.1 | 4676 | 28.0 | 4066 | 42.8 | 705 | 9.8 |
| 50 – | 11.7 | 6.9 | 4.8 | 6516 | 55.7 | 5216 | 75.6 | 1238 | 25.8 | 7628 | 65.2 | 6216 | 90.1 | 1521 | 31.7 |
| 60 – | 6.5 | 3.5 | 3.0 | 5349 | 82.3 | 4448 | 127.1 | 1341 | 44.7 | 6454 | 99.3 | 5444 | 155.5 | 1713 | 57.1 |
| 70 – | 2.4 | 1.1 | 1.3 | 2215 | 92.3 | 1513 | 137.5 | 829 | 63.8 | 3864 | 161.0 | 2092 | 190.2 | 1257 | 96.7 |
| 80 – | 0.6 | 0.2 | 0.4 | 449 | 74.8 | 253 | 126.5 | 225 | 56.3 | 1147 | 191.2 | 560 | 280.0 | 633 | 158.3 |
| Total | 161.4 | 84.6 | 76.8 | 21257 | | 16850 | | 4787 | | 26429 | | 20575 | | 6453 | |

*International comparison*

The age-adjusted death rates for hepatoma and cirrhosis of the liver of selected countries are shown in Tables II and III respectively. Apparently the death rates for both hepatoma and cirrhosis of the liver in Taiwan belong at, or near the top among those countries for both males and females.

**Table II** Age-adjusted death rates from hepatoma in 11 selected countries by sex (1979–1981)

| *Country* | *Male* | *Female* |
|---|---|---|
| *Taiwan* | 26.10 | 8.14 |
| Japan | 10.77 | 3.12 |
| Austria | 3.28 | 1.53 |
| Singapore | 2.74 | 1.43 |
| Israel | 2.44 | 0.72 |
| France | 2.33 | 0.68 |
| Chile | 2.30 | 1.46 |
| Germany (Fed. Rep. of) | 1.56 | 0.63 |
| USA | 1.23 | 0.41 |
| UK | 1.22 | 0.34 |
| Netherlands | 1.09 | 0.32 |

Rates are numbers of deaths per 100,000 population and adjusted to the WHO standard population.

**Table III** Age-adjusted death rates of cirrhosis of liver in 20 selected countries by sex (1978–1980)

| *Country* | *Male* | *Female* |
|---|---|---|
| Chile | 60.78 | 22.23 |
| Italy | 33.42 | 10.36 |
| *Taiwan* | 33.01 | 12.90 |
| France | 32.28 | 12.72 |
| Austria | 31.17 | 8.65 |
| Germany | 25.73 | 8.75 |
| Mauritius | 23.41 | 3.44 |
| Egypt | 17.29 | 7.07 |
| Japan | 16.01 | 4.42 |
| USA | 15.99 | 7.34 |
| Canada | 15.15 | 6.32 |
| Switzerland | 12.98 | 3.54 |
| Denmark | 11.66 | 4.08 |
| Sweden | 10.74 | 4.54 |
| Thailand | 10.13 | 3.27 |
| Israel | 9.95 | 3.62 |
| Philippines | 9.32 | 3.22 |
| Singapore | 8.98 | 3.28 |
| Netherlands | 5.37 | 2.06 |
| UK | 2.99 | 2.17 |

Rates are numbers of deaths per 100,000 population and adjusted to the WHO standard population.

**Figure 4** Map of the age-adjusted death rates of hepatoma in Taiwan by areas (1971–1980). ■ Significantly higher than Taiwan area. 19.7–30.4/100,000. ▨ Not significant. 18.0–19.6/100,000. □ Significantly lower than Taiwan area. 11.4–16.6/100,000.

**Figure 5** Map of the age-adjusted death rates of cirrhosis of liver in Taiwan by area (1971–1980). ■ Significantly higher than Taiwan area. 26.1–36.2/100,000. ⊡ Not significant. 24.1–25.3/100,000. □ Significantly lower than Taiwan area. 18.3–21.9/100,000.

### Discussion

In reviewing the international mortality of hepatoma and cirrhosis of liver, it is evident that the death rates of these two liver diseases in Taiwan are among the highest in the world. Because of the high case-fatality rate and short duration of the disease process, especially for hepatoma, the death rates may also represent the incidence rates. An interesting observation in this study is that while the secular trend of the death rates of hepatoma has been increasing for males, virtually no change of the rates for females is noted over the time period. For cirrhosis of the liver, though increases in the death rates are observed for both sexes, the upward trend is more remarkable for males. Accordingly the secular trends of sex ratios of mortality from hepatoma and cirrhosis of liver have been increasing. The increasing death rates may be partly due to the improvement of diagnosis. However, the more rapid increase for males than for females cannot be explained only by the improvement of diagnosis. Saracci and Repetto (1980) reported that among 30 selected cancer registries covering 37 populations in 18 countries the trends in incidence of primary liver cancer (PLC) are similar in males and females. However, the sex ratios of PLC in most of these areas show definite male preponderance. The large difference of mortality rates of hepatoma and cirrhosis of liver between males and females and the increasing trends of sex ratios of mortality of these two diseases suggest that factors in addition to HBV are important in the aetiology of these diseases because similar rates of HBV infection and carrier status have been reported in Taiwan for the two sexes and more than 80% of hepatomas and cirrhosis of the liver in Taiwan have been found to be permanent carriers of HBV (Chen *et al.*, 1978; Wu *et al.*, 1980).

Several reasons can be considered for the increasing trends of sex ratios in mortality from hepatoma and cirrhosis of liver in Taiwan, among which the reliability of the data seems most critical. Though the registration of death is virtually universal in Taiwan, the exactness of the diagnosis of causes of death is, of course, as in all countries subject to some uncertainty. Recent studies conducted by the National Health Administration in Taiwan (1979–80) found that although there are very few autopsies performed, about 40% of hepatomas were histologically confirmed by biopsy, which compares favourably with histological confirmation rates for this neoplasm in various cancer registries throughout the world. Furthermore, most cases of hepatoma also have cirrhosis so that there is substantial room for misdiagnosis as well as overlap between these two disease entities. The difficulty of diagnosing hepatoma and cirrhosis of liver makes the reliability of the data uncertain to an unknown extent especially since these are based on death certificates. However, it should be noted that most cirrhosis in Taiwan is macronodular which is most frequently due to HBV infection rather than micronodular variety which is more associated with excess alcohol consumption. Finally it may be noted that though the present study combined cancer of the liver and cancer of the intrahepatic bile duct (ICD 155), more than 95% of the cases in Taiwan are of cancer of the liver.

The large difference in mortality rates for hepatoma and cirrhosis of the liver between males and females as well as the increasing trends of the sex ratios in Taiwan, however, may suggest that besides HBV antigenemia, other factors may be involved in the aetiology of these diseases. The reasons why HBsAg disappears in most patients with HBV hepatitis while a minority of patients become persistent carriers of HBsAg have not been elucidated, but may relate to either viral or host factors. The bulk of evidence, however, suggests that host factors are more important in the development of the chronic carrier state. Blumberg *et al.* (1969) first reported an autosomal recessive inheritance of susceptibility to infection of HBV and suggested that genetic factors are involved in those who become carriers, though subsequent data from several studies are inconsistent with such inheritance. (Vyas, 1974; Szmuness *et al.*, 1975; Stevens & Beasley, 1976). Holland & Alter (1976) also suggested that host factors are more important in the development of the chronic carrier state than viral factors. Renal dialysis patients, when infected, tend to develop mild clinical disease and a persistent carrier state, whereas the staff presumably infected with the same virus, develop acute, often severe disease, but no predisposition to have chronic antigenemia. Moreover, the observation of high levels of HBsAg in asymptomatic persons otherwise free of liver diseases also favours a host factor. In addition to those with renal disease, patients with leukaemia, Down's syndrome and lepromatous leprosy also have exhibited a striking tendency to develop chronic HBsAg carrier status. (Blumberg *et al.*, 1967).

The association of HBV with hepatoma and cirrhosis of liver has been documented by many workers (Steiner, *et al.*, 1960; Szmuness *et al.*, 1978; Tabor *et al.*, 1977; Summer *et al.*, 1978; Beasley *et al.*, 1981). The following facts have been included: a high prevalence of HBsAg in hepatoma and patients with cirrhosis of liver, a high incidence of hepatoma and cirrhosis of liver among HBsAg carriers, and the detection of HBV genomes in hepatoma cells and animal models. However, other environmental factors may be involved in the aetiology of hepatoma and cirrhosis of liver. Aflatoxin may act synergistically or even independently of HBV in the causation of hepatoma. In Taiwan the contamination of foodstuff with aflatoxins is a serious problem (Tung *et al.*, 1967; Ling *et al.*, 1967; Ling *et al.*, 1968). It is considered that the high incidence of PLC in Taiwan may be explained partly by aflatoxin (Tung *et al.*, 1967). Van Rensburg *et al.* (1985) suggested that an interaction between HBV and aflatoxin may be responsible for the exceptionally high rates of hepatocellular carcinoma (HCC) evident in parts of Africa and Asia and indicated that aflatoxin has a late stage effect on the development of HCC. Alcohol is considered to be one of the aetiological factors for hepatoma in some countries and the major one for cirrhosis in many parts of the world. Alcohol consumption and chronic alcoholism are known to be much less common in Chinese than other ethnic groups and constitute a minor social and medical problem in Taiwan. It is especially noteworthy that there is very little alcohol consumption among Chinese women. In a case-control study conducted recently by the authors, neither alcohol consumption nor duration and/or drinking frequency were found to be significantly associated with hepatoma. (Lin *et al.*, to be published). Malnutrition, malaria and intestinal parasites may be co-factors in some countries but in Taiwan both malnutrition and malaria have been almost nonexistent for more than 25 years. It is suggested that the possible role of factors additional to HBV should be investigated to clarify the remarkable sex difference and the increasing trends of sex ratios in mortality rates of hepatoma and cirrhosis of liver in Taiwan.

The authors are grateful to Dr R.P. Beasley, University of Washington Medical Research Unit, and Dr R.S. Lin, National Taiwan University College of Medicine for their suggestions and reading this manuscript. Thanks are due to Mr T.S. Tsai for technical assistance.

### References

BEASLEY, P., HWANG, L.Y., LIN, C.C. & CHIEN, C.S. (1981). Hepatocellular carcinoma and hepatitis B virus. A prospective study of 22707 men in Taiwan. *Lancet*, **ii**, 1129.

BLUMBERG, B.S., GERSTLEY, B.J., HUNGERFORD, D.A., LONDON, W.T. & SUTNICK, A.I. (1967). A serum antigen (Australia) in Down's syndrome, leukemia and hepatitis. *Ann. Int. Med.*, **66**, 924.

BLUMBERG, B.S., FRIEDLANDER, J.S., WOODSIDE, A. SUTNICK, A.I. & LONDON, W.T. (1969). Hepatitis and Australia antigen: autosomal recessive inheritance of suceptibility to infection in human. *Proc. Natl Acad. Sci.*, **62**, 1108.

CHEN, D.S., SUNG, J.L. & LAI, M.Y. (1978). A seroepidemiologic study of hepatitis B virus infection in Taiwan. *J. Form. Med. Assoc.*, **77**, 908.

HOLLAND, P.V. & ALTER, M.J. (1976). Current concepts of viral hepatitis. In *Viral Infections. A Clinical Approach*, p. 189. F.A. Davis Company: San Francisco.

LIN, T.M., LU, S.N., CHEN, C.J., LIAU, Y.F. & CHANG, W.Y. (1986). Multiple risk factors of primary hepatocellular carcinoma in Taiwan. A matched case-control study. (In press).

LING, K.H., WANG, J.J., WU, R. & 4 others. (1967). Intoxication possibly caused by aflatoxin B1 in the moldy rice in Shuangchih Township. *J. Form. Med. Assoc.*, **66**, 517.

LING, K.H., TUNG, C.M., SHEH, I.F., WANG, J.J. & TUNG, T.C. (1968). Aflatoxin B in unrefined peanut oil and peanut products in Taiwan. *J. Form. Med. Assoc.*, **67**, 309.

NATIONAL HEALTH ADMINISTRATION. *The Executive Yuan*. ROC (1979–1980). Cancer registry annual report in Taiwan area.

REPUBLIC OF CHINA NATIONAL HEALTH ADMINISTRATION (1974–80). *Health Statistics*.

SARACCI, R. & REPETTO F. (1980). Time trend of primary liver cancer. Indication of increased incidence in selected cancer registry population. *J. Natl Cancer Inst.*, **65**, 241.

STEINER, P.E. (1960). Cancer of the liver and cirrhosis in trans-Saharan Africa and the United States of America. *Cancer*, **13**, 1085.

STEVENS, C.E. & BEASLEY, R.P. (1976). Lack of an autosomal recessive genetic influence in vertical transmission of hepatitis B antigen. *Nature*, **260**, 715.

SUMMERS, J., O'CONNELL, A. & MAUPAS, P. (1978). Hepatitis B virus DNA in primary hepatocellular carcinoma tissue. *J. Med. Virol.*, **2**, 207.

SZMUNESS, W., HARLEY, E.I. & PRINCE, A.M. (1975). Intrafamiliar spread of asymptomatic hepatitis B. *Am. J. Med. Sci.*, **270**, 293.

SZMUNESS, W. (1978). Hepatocellular carcinoma and hepatitis B virus. Evidence for causal association. *Prog. Med. Virol.*, **24**, 40.

TABOR, E., GARETZ, R.T. & VAGEL, C.J. (1977). Hepatitis B virus infection and primary hepatocellular carcinoma tissue. *J. Med. Virol.*, **2**, 207.

TAIWAN PROVINCIAL GOVERNMENT DEPARTMENT OF HEALTH (1971–73). *Vital Statistical Abstract*.

TUNG, T.C., SHIAU, C.C., LO, J.T. & LIN, K.H. (1967). Survey of foodstuff in Taiwan for the aflatoxin producing strain of aspergillus flavus. *J. Form. Med. Assoc.*, **66**, 339.

VAN RENSBURG, S.J., COOK-MOZAFFARI, P., VAN SCHALKWYK, D.J. & 3 others. (1965). Hepatocellular carcinoma and dietary aflatoxin in Mozambique and Transkei. *Br. J. Cancer.*, **51**, 713.

VYAS, G.N. (1974). Evidence against recessive inheritance of suceptibility to the chronic carrier state for hepatitis B antigen. *Nature,* **248,** 159.

WORLD HEALTH ORGANIZATION (1965). *International Classification of Diseases, Injuries, and Causes of Death.* 8th Revision.

WORLD HEALTH ORGANIZATION (1971–80). *Vital Statistics and Causes of Death.*

WU, H.Y., CHEN, K.P., TSENG, W.P. & HSU, C.L. (1981). Epidemiologic studies on Blackfoot disease. 1. Prevalence and incidence of the disease by age, sex, year, occupation and geographic distribution. *Mem. Coll. Med. Nat. Taiwan Univ.,* **VII,** 33.

WU, J.S., CHEN, C.H., CHIANG, Y.H. & 4 others. (1980). Hepatitis B virus infection in Taiwan with reference to anti-HBc versus HBsAg and anti-HBs. *J. Form. Med. Assoc.,* **79,** 760.